# Quality of life measures in treatment for active substance misuse: a scoping review

**DOI:** 10.1007/s11136-025-04072-0

**Published:** 2025-10-16

**Authors:** Anna L. Milliken, Kimberly O. Collamore, Stacy R. Ryan-Pettes

**Affiliations:** Department of Psychology and Neuroscience, Baylor University, One Bear Place 97334, Waco, TX 76798 USA

**Keywords:** Quality of life, Health-related quality of life, Quality of life measures, Substance use disorder, Substance use disorder treatment

## Abstract

**Purpose:**

Unlike previous reviews on quality of life (QoL) and substance use disorders (SUD), this scoping review focuses on QoL within the context of SUD treatment and active substance misuse. This review delineates and describes QoL measures, assesses their application, and explores the influence of demographics on QoL outcomes.

**Methods:**

Five databases were searched (EMBASE, Web of Science, PsycINFO, PubMed, and Scopus) using keywords related to QoL, clinician-verified SUD, and SUD treatment. All studies were published in English before June 1st, 2025. Two reviewers screened abstracts and full text for eligibility and study elements. The first author extracted data regarding methodology, substance use, treatment, and QoL measures. The PRISMA-ScR checklist guided this study and the reporting process for rigor and transparency.

**Results:**

Of the 11,078 initially identified articles, 108 were extracted for analysis. QoL measures accounted for multiple domains (e.g., physical, psychological, social, environmental, independence, security, and spiritual). Studies included male and female participants, all over the age of 18. Results found that many studies under-reported race, ethnicity, and cultural contexts, as well as high variability in the definition of QoL domains and standardization across treatment locations and geographic regions. Studies from Asia primarily showed preference for the WHOQOL-BREF measure compared to North America and Europe, which showed varied preference for measures. The most common QoL domains assessed were psychological, physical, and social.

**Conclusion:**

This review highlights the need to operationalize QoL domains and assessment approaches in SUD treatment research to facilitate meaningful comparisons and enhance our understanding of treatment effectiveness.

## Introduction

Recent estimates show that in the United States, 21.4% of adults and 12.8% of adolescents reported using illicit substances in the past year [1], with over 48.5 million people over the age of 12 (approximately 17%) diagnosed with a substance use disorder in the past year [2]. Substance use disorders (SUD), defined as substance use despite harmful consequences [3], are associated with physical complications, psychological impairment, social isolation, and financial instability [4], often requiring multiple treatment episodes to achieve and sustain recovery [3].

SUD treatment is often conceptualized as acute and time-limited, lasting from several weeks to several months [5]. However, there is substantial variability in treatment structure and duration based on individual factors (e.g., individual needs, severity of substance use disorder, co-occurring medical or mental conditions, treatment setting, and accessibility of resources). For example, residential or inpatient treatment settings generally provide more medically based, structured, and short-term treatment compared to outpatient treatment, which may include contingency management of psychotherapy ([6, 7]. This heterogeneity in treatment often complicates operationalization of SUD treatment in research and opens the door to exploring how to operationalize changes, including meaningful changes in QoL. Additionally, variability in treatment duration can range from brief to intensive episodes of care, depending on the severity of use and individual patient needs, resulting in substantial differences in the operationalization of optimal outcome monitoring [7].

Recent research emphasizes the importance of measuring non-abstinence-focused approaches to recovery, such as improvement in quality of life (QoL) in SUD treatment [8]. QoL is defined as an individual’s subjective ratings of overall well-being across social, physical, and psychological domains and has been increasingly used as an outcome measure of treatment effectiveness and in identifying intervention targets in SUD treatment [9]. Utilizing QoL provides a more holistic assessment that extends beyond substance use reduction to assess broader domains of functioning and well-being [10]. QoL encompasses several key domains: physical health (e.g., energy, pain, general health), psychological well-being (e.g., mental health, cognition, memory), social relationships (e.g., relationship quality, social support), environmental factors (e.g., work environment, living conditions, healthcare access), and spiritual meaning [11]. Within healthcare settings, a subset known as Health-Related QoL (HRQoL) focuses specifically on the interactions between health conditions and medical interventions, emphasizing factors such as the ability to perform daily activities and limitations due to pain [12], and is commonly applied in clinical research as an evaluative tool for medical interventions compared to general QoL (G-QoL) [13].

Recent research demonstrates that individuals with SUD experience significantly lower QoL compared to those with other psychiatric disorders [14]. Additionally, participants report QoL concerns beyond maintaining abstinence, such as housing circumstances, employment, and discrimination [15]. Despite growing recognition of the importance of evaluating QoL in substance use treatment, there is a significant gap in understanding how these measures are used and applied in SUD treatment research. While several QoL measures exist, their application lacks consistency, and their association with meaningful SUD treatment outcomes remains unclear, especially for marginalized communities that may experience additional psychosocial stressors [16].

### Purpose of current study

This systematic scoping review addresses these gaps by synthesizing the current evidence on QoL measurement in SUD treatment to inform future research. While existing reviews have established the general association between QoL and SUD [17], a critical gap remains in understanding how QoL measures are operationalized and applied in SUD treatment. This scoping review aims to (1) characterize the types of QoL measures used in SUD treatment research, including how these measures are defined and operationalized; (2) Examine how QoL domains are conceptualized and measured across different treatment settings (inpatient, outpatient, during treatment, after treatment) and populations; (3) identify how patterns in how demographic factors, and cultural context influence QoL measurement in SUD treatment; and (4) Describe trends in QoL measurement approaches from 2000–2025 to understand how the field has evolved.

## Method

### Search strategy

The current review followed the 2020 Preferred Reporting Items for Systematic Review and Meta-Analyses Extension for Scoping Reviews (PRISMA-ScR) [18]. Two independent investigators searched the following databases from inception to October 31, 2023: EMBASE, Web of Science, PsycINFO, PubMed, and Scopus using relevant terms related to “QOL” and “SUD” (see Table 1 for search terms). Article management (e.g., importing sources, removing duplicates, title/abstract screening, and data extraction) was performed using Covidence software [19].

**Table 1:** Search strategy

Search Terms
(“quality of life” or “health related quality of life” or “quality of life measure”) AND
(“substance abuse” or “substance use” or “drug abuse” or “addiction” or “drug use” or “drug dependence” or “substance related and addictive disorders" or "cannabis use disorder" or "opioid use disorder" or "tobacco use disorder" or "drug addiction" or "alcohol use disorder" or "polydrug abuse")

The same search terms were used for EMBASE, Web of Science, PsycINFO, PubMed, and Scopus

### Study selection

Articles were considered for inclusion if: (1) studies involved subjects with a SUD diagnosed through the Diagnostic and Statistical Manual of Mental Disorders, Fourth Edition (DSM-IV), DSM, Fifth Edition (DSM-V) or the International Classification of Disease (ICD) criteria (2) participants received any treatment for active substance misuse, (3) studies reported use of a standardized measure of QoL, (4) studies included any quantitative data of the association between SUD treatment and QoL, and (5) studies were published in English*.*

Studies were excluded if they were (1) case studies, book chapters, dissertations/theses, and congressional reports, (2) unpublished, and (3) published before the year 2000 as diagnostic criteria, treatment models, and measurement approaches have evolved since the publication of the DSM-IV-TR and subsequent revisions have shaped conceptualization of SUD.

### Selection of studies

**Fig. 1:**
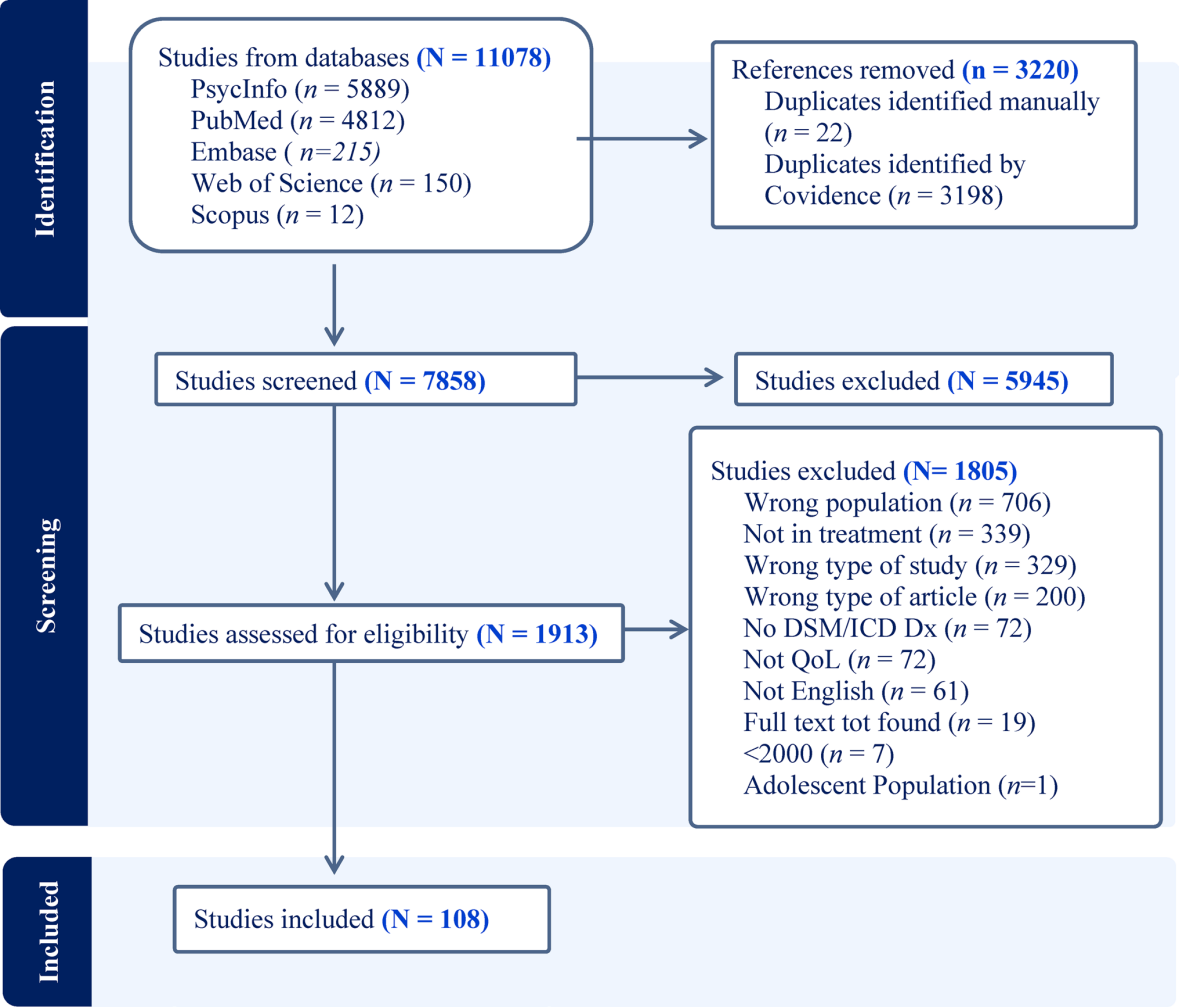
Summary of search methods

From the initial 7,858 studies identified through database searches, 1,865 studies were moved to full-text review after title and abstract screening. Of these, 1,913 were excluded based on the selection criteria. Among all articles reviewed, only one article included participants under 18 and was removed from the current review due to a lack of generalizability compared to the adult populations. Six additional studies were merged to combine multiple references reporting the same results into a single study record. One hundred and eight studies were included in the current review. The first and second authors conducted the full-text review, and the interrater reliability was *strong* (Cohen’s Kappa = . 81) [20]. See Fig. 1 for the number of articles identified and excluded at each step.

### Data extraction

The first and second authors extracted data from the included studies (N = 108). The first author extracted data from all included articles, while the second author extracted data for 30% of the included articles (*n* = 30), and the interrater reliability was *strong* (Cohen’s Kappa = .71) [20}]. The following data were collected on study participants and the research design: country of origin, study type, study aim, inclusion and exclusion criteria, mean age/age range, gender, majority racial/ethnic composition of the study, total number of participants, and comorbid mental and medical conditions. For the substance use variables, the following data were collected: setting of SUD treatment (e.g., inpatient, outpatient, residential treatment centers), the substance used, criteria for SUD (e.g., DSM, ICD), and type of treatment (e.g., medical detox/medication management, psychotherapy, contingency management, exercise interventions, and a combination of medical assistance and psychotherapy). The following data were collected on the QoL outcome variable: type of QoL assessed, measures used to assess QoL, how the measure was used (e.g., prediction, outcome) if there was baseline and follow-up testing on QoL (i.e., yes, no), if there were group comparisons between QoL (i.e., treatment group, mental health, type of substance use, treatment location, other comparisons), and broad domains and individual facets of QoL, as well as results trends related to QoL.

### Quality assessment

To assess methodological quality and risk of bias, the first and second authors conducted a quality assessment using the National Institute of Health (NIH) quality assessment tool. The first author conducted a quality assessment of all the articles included. The second author completed the evaluation for 30% of the articles included (*n* = 30), and the interrater reliability was *strong* (Cohen’s Kappa = . 78) [20]. Since this scoping review contained many different studies, the first and second authors used the quality assessment tool for Observational Cohort and Cross-Sectional Studies and the assessment tool for randomized clinical control trials [21]. To ensure consistency in quality and risk assessment, each article was referenced to 14 questions regarding methodological clarity and outcomes. Each item was rated “no,” “yes,” “not applicable,” or “not reported.” The number of “yes” + “not applicable” and “no” + “not reported” ratings for each study were aggregated to yield an overall quality rating and classified as "good," "fair," or "poor."

## Results

### Overview of included studies

Table 2 provides demographic characteristics and results from each study. The final sample included 108 [22–130] studies published between 2000 and 2025. Most studies were conducted in Europe (43.51%) and North America (32.41%), with fewer from Asia (14.81%) and other regions, including Australia (6.48%), Africa (1.85%), and South America (1.85%). Most studies included both female and male participants (83.33%), with approximately 11.11% including only male participants and 5.56% including only female participants. No articles provided information regarding clients who held other gender identities (e.g., transgender, non-binary). However, reporting on age and racial/ethnic demographics was limited. Twenty-three (20.37%) studies reported age ranges (total range:18–73 years), and five (4.85%) studies did not include information on the age of the adult participants. Additionally, seventy-eight studies (72.22%) failed to provide data on racial/ethnic demographics. Of the studies that reported on race/ethnicity *(*n = 20), most participants were White. The sample size ranged from 29 to 2176 participants in each study. Most studies (67.59%) used G-QoL measures, 31.48% used HRQoL measures, and a small fraction (0.93%) used both. Three primary QoL measures emerged as predominant in SUD treatment research: the WHOQOL-BREF (*n* = 37, 34.26%) [131], SF-36 (*n* = 23, 21.30%) [132] and EQ-5D *(n* = 16, 14.81%) [133]. For full psychometric considerations of QoL measures, please see supplemental material.

**Table 2:** Demographic and characteristics of included articles

*Characteristics*	Full Sample N = 108		G-QOL *n* = *73*		HR-QOL *n* = 34		Inpatient treatment *n* = 28		Outpatient treatment *n* = 63	
	N	%	n	%	n	%	n	%	n	%
*Country*										
United States	30	27.78	22	30.14	8	23.53	3	10.71	19	30.16
Australia	6	5.56	5	6.85	1	2.94	1	3.57	3	4.76
Norway	6	5.56	4	5.48	2	5.88	2	7.14	4	6.35
Turkey	6	5.56	5	6.85	1	2.94	4	14.29	1	1.59
Germany	5	4.63	2	2.74	3	8.82	3	10.71	2	3.17
Spain	5	4.63	2	2.74	3	8.82	0	0.00	5	7.94
Netherlands	4	3.70	1	1.37	2	5.88	0	0.00	4	6.35
Finland	3	2.78	3	4.11	0	0.00	1	3.57	2	3.17
India	5	4.63	5	6.85	0	0.00	3	10.71	0	0.00
Sweden	3	2.78	0	0.00	3	8.82	0	0.00	2	3.17
Other^a^	35	32.41	24	32.88	11	32.35	11	39.29	21	33.33
Sex										
Both	90	83.33	55	75.34	34	100.00	24	85.71	54	85.71
Male	12	11.11	12	16.44	0	0.00	3	10.71	7	11.11
Female	6	5.56	6	8.22	0	0.00	1	3.57	2	3.17
Race										
Assessed	30	27.78	21	28.77	8	23.53	5	17.86	20	31.75
*Treatment design*										
Randomized controlled trial	49	45.37	32	43.84	16	47.06	2	7.14	36	57.14
Pre/Post data	34	69.39	21	65.63	12	75.00	2	100.00	24	66.67
Baseline & follow up	33	97.06	20	95.24	12	100.00	1	50.00	24	100.00
Baseline data only	1	2.94	1	4.76	0	0.00	1	50.00	0	0.00
Comparisons (yes)	33	67.35	21	65.63	11	68.75	2	100.00	21	58.33
Treatment	24	72.73	15	71.43	9	81.82	1	50.00	16	76.19
Substance	4	12.12	2	9.52	1	9.09	0	0.00	3	14.29
Other^b^	3	9.09	2	9.52	1	9.09	1	50.00	2	9.52
Mental health diagnosis	2	6.06	2	9.52	0	0.00	0	0.00	0	0.00
Significance in QoL	38	77.55	27	84.38	10	62.50	2	100.00	27	75.00
Between Time Points	20	52.63	16	59.26	4	40.00	2	100.00	15	55.56
Between Study Groups	13	34.21	7	25.93	5	50.00	0	0.00	10	37.04
Within Covariates	5	13.16	4	14.81	1	10.00	0	0.00	2	7.41
Cross Sectional	31	28.70	26	35.62	5	14.71	14	50.00	14	22.22
Pre/Post data	7	22.58	7	26.92	0	0.00	3	21.43	3	21.43
Both Baseline & Follow up	7	100.00	7	100.00	0	#DIV/0!	3	100.00	3	100.00
Baseline data only	0	0.00	0	0.00	0	#DIV/0!	0	0.00	0	0.00
Comparisons (yes)	16	51.61	15	57.69	1	20.00	7	50.00	7	50.00
Treatment	5	31.25	5	33.33	0	0.00	2	28.57	3	42.86
Substance	4	25.00	4	26.67	0	0.00	1	14.29	2	28.57
Other^c^	4	25.00	4	26.67	0	0.00	3	42.86	0	0.00
Mental health diagnosis	3	18.75	2	13.33	1	100.00	1	14.29	2	28.57
Significance in QoL	27	87.10	22	84.62	5	100.00	12	85.71	14	100.00
Between Treatment Groups	13	48.15	12	54.55	1	20.00	7	58.33	5	35.71
Between Covariates	9	33.33	5	22.73	4	80.00	3	25.00	6	42.86
Between Time Points	5	18.52	5	22.73	0	0.00	2	16.67	3	21.43
Cohort Study	15	13.89	8	10.96	7	20.59	8	28.57	6	9.52
Pre/Post data	6	40.00	5	62.50	1	14.29	2	25.00	3	50.00
Both Baseline & Follow up	5	83.33	4	80.00	1	100.00	1	50.00	3	100.00
Baseline data only	1	16.67	1	20.00	0	0.00	1	50.00	0	0.00
Comparisons (yes)	6	40.00	4	50.00	2	28.57	3	37.50	3	50.00
Treatment	2	33.33	1	25.00	1	50.00	1	33.33	1	33.33
Mental health diagnosis	2	33.33	2	50.00	0	0.00	2	66.67	0	0.00
Other^b^	2	33.33	1	25.00	1	50.00	0	0.00	2	66.67
Substance	0	0.00	0	0.00	0	0.00	0	0.00	0	0.00
Significance in QoL	10	66.67	6	75.00	4	57.14	5	62.50	4	66.67
Between Time Points	4	40.00	2	33.33	2	50.00	2	40.00	2	50.00
Between Covariates	3	30.00	2	33.33	1	25.00	3	60.00	0	0.00
Between Treatment Groups	3	30.00	2	33.33	1	25.00	0	0.00	2	50.00
Non-Randomized Experiment	5	4.63	2	2.74	3	8.82	3	10.71	2	3.17
Pre/Post data	5	100.00	2	100.00	3	100.00	3	100.00	2	100.00
Both Baseline & Follow up	5	100.00	2	100.00	3	100.00	3	100.00	2	100.00
Baseline data only	0	0.00	0	0.00	0	0.00	0	0.00	0	0.00
Comparisons (yes)	4	80.00	2	100.00	2	66.67	2	66.67	2	100.00
Treatment	3	75.00	2	100.00	1	50.00	1	50.00	2	100.00
Other^b^	1	25.00	0	0.00	1	50.00	1	50.00	0	0.00
Substance	0	0.00	0	0.00	0	0.00	0	0.00	0	0.00
Mental health diagnosis	0	0.00	0	0.00	0	0.00	0	0.00	0	0.00
Significance in QoL	5	100.00	2	100.00	3	100.00	3	100.00	2	100.00
Between Time Points	5	100.00	2	100.00	3	100.00	3	100.00	2	100.00
Between Covariates	0	0.00	0	0.00	0	0.00	0	0.00	0	0.00
Between Treatment Groups	0	0.00	0	0.00	0	0.00	0	0.00	0	0.00
Other^c^	8	7.41	5	6.85	3	8.82	1	3.57	5	7.94
Pre/Post data	3	37.50	2	40.00	1	33.33	0	0.00	2	40.00
Both Baseline & Follow up	3	100.00	2	100.00	1	100.00	0	0.00	2	100.00
Baseline data only	0	0.00	0	0.00	0	0.00	0	0.00	0	0.00
Comparisons (yes)	6	75.00	5	100.00	1	33.33	1	100.00	3	60.00
Treatment	4	66.67	3	60.00	1	100.00	0	0.00	3	100.00
Substance	2	33.33	2	40.00	0	0.00	1	100.00	0	0.00
Mental health diagnosis	0	0.00	0	0.00	0	0.00	0	0.00	0	0.00
Other^b^	0	0.00	0	0.00	0	0.00	0	0.00	0	0.00
Significance in QoL	6	75.00	3	60.00	3	100.00	1	100.00	4	80.00
Between Covariates	3	50.00	1	33.33	2	66.67	1	100.00	2	50.00
Between Time Points	2	33.33	1	33.33	1	33.33	0	0.00	2	50.00
Between Treatment Groups	1	16.67	1	33.33	0	0.00	0	0.00	0	0.00
*Substance Use & Treatment*										
Substance										
Multiple Substances	36	33.33	29	39.73	6	17.65	10	35.71	17	26.98
Alcohol	33	30.56	23	31.51	15	44.12	14	50.00	20	31.75
Opioid	30	27.78	17	23.29	12	35.29	4	14.29	22	34.92
Other^d^	2	1.85	2	2.74	0	0.00	0	0.00	2	3.17
Stimulant	2	1.85	1	1.37	1	2.94	0	0.00	1	1.59
SUD diagnosis Method										
DSM-IV	66	61.11	46	63.01	19	55.88	15	53.57	37	58.73
ICD-10	25	23.15	14	19.18	11	32.35	8	28.57	15	23.81
DSM-V	12	11.11	8	10.96	4	11.76	3	10.71	8	12.70
Location										
Outpatient	63	58.33	41	56.16	21	61.76	0	0.00	63	100.00
Inpatient	28	25.93	21	28.77	7	20.59	28	100.00	0	0.00
Multiple treatment locations	11	10.19	8	10.96	3	8.82	0	0.00	0	0.00
Residential	6	5.56	3	4.11	3	8.82	0	0.00	0	0.00
Type of treatment										
Medical Detox/ Medication	63	58.33	39	53.42	23	67.65	21	75.00	33	52.38
Medical Detox & Psychotherapy	21	19.44	17	23.29	4	11.76	4	14.29	11	17.46
Psychotherapy	18	16.67	13	17.81	5	14.71	0	0.00	17	26.98
Contingency Management	2	1.85	2	2.74	0	0.00	0	0.00	1	1.59
Exercise Treatment	2	1.85	0	0.00	2	5.88	1	3.57	1	1.59
Total Number of Mental Health Dx										
Not mentioned/ Not assessed	68	62.96	43	58.90	25	73.53	15	53.57	40	63.49
1–2 Conditions	20	18.52	15	20.55	5	14.71	7	25.00	10	15.87
3–5 conditions	7	6.48	6	8.22	1	2.94	4	14.29	3	4.76
> 5 Conditions	13	12.04	9	12.33	3	8.82	2	7.14	10	15.87
Total Number of Medical Dx										
Not mentioned/ Not assessed	76	70.37	52	71.23	23	67.65	22	78.57	41	65.08
1–2 Conditions	19	17.59	13	17.81	6	17.65	4	14.29	15	23.81
3–5 conditions	11	10.19	6	8.22	5	14.71	1	3.57	6	9.52
> 6 Conditions	2	1.85	2	2.74	0	0.00	1	3.57	1	1.59
*QoL*										
QoL Measure										
G-QoL	73	67.59	73	100.00	34	100.00	21	75.00	41	65.08
HR-QoL	34	31.48	0	0.00	0	0.00	7	25.00	21	33.33
Both	1	0.93	0	0.00	0	0.00	0	0.00	1	1.59
QoL Measure										
WHOQOL-BREF	37	34.26	37	50.68	0	0.00	14	50.00	19	30.16
SF-36	23	21.30	3	4.11	18	52.94	5	17.86	11	17.46
EQ-5D	16	14.81	6	8.22	7	20.59	0	0.00	11	17.46
SF-12	7	6.48	1	1.37	5	14.71	1	3.57	5	7.94
Q-LES-Q	8	7.41	8	10.96	0	0.00	0	0.00	5	7.94
QOLI	4	3.70	4	5.48	0	0.00	0	0.00	3	4.76
MSQoL	2	1.85	0	0.00	2	5.88	1	3.57	1	1.59
KQL	2	1.85	0	0.00	0	0.00	0	0.00	0	0.00
SQOL	1	0.93	1	1.37	0	0.00	1	3.57	0	0.00
BASIS-32	1	0.93	1	1.37	0	0.00	1	3.57	0	0.00
SQLS	1	0.93	1	1.37	0	0.00	1	3.57	0	0.00
SDS	1	0.93	0	0.00	1	2.94	0	0.00	1	1.59
FAHI	1	0.93	1	1.37	0	0.00	0	0.00	1	1.59
QLQ	1	0.93	1	1.37	0	0.00	0	0.00	1	1.59
LQLP	1	0.93	1	1.37	0	0.00	0	0.00	1	1.59
*Quality assessment*										
Good	55	50.93	35	47.95	19	55.88	12	42.86	33	52.38
Fair	51	47.22	37	50.68	14	41.18	16	57.14	28	44.44
Poor	2	1.85	1	1.37	1	2.94	0	0.00	2	3.17

Specified percentages are as follows: *indicates taken out of sample for the treatment design, ** indicates taken out of the subheading under the treatment design

^a^Other countries include Austria, Belgium, Bosnia, Brazil, Canada, Croatia, Czech Rep., Denmark, Egypt, France, Italy, Lebanon, Malaysia, Poland, Puerto Rico, Iran, Israel, and South Africa

^b^Other types of group comparisons include medical condition, gender, military status, study completers vs. non-completers, stigma, different studies, and Treatment Location)

^c^Other types of studies include Prospective Observational Study, Matrix Intervention, and Mediation Analysis

^d^Tobacco, Cannabis, Benzodiazepine, or not specified

### Aim 1: Qol measures in SUD treatment research

#### 1Methodological characteristics

The most common study designs were randomized control trials (45.37%) and cross-sectional studies (28.70%). Half of the included (50.00%) contained both baseline and follow-up QoL assessments, all of which included measurement of QoL at baseline and follow-up assessment points, with follow-up assessments ranging from 3 weeks to 3 years post-treatment. Most studies were conducted in outpatient settings (58.33%), with medical detoxification and medication management as the primary treatment approaches (58.33%). Studies primarily focused on multiple substance use (33.33%), alcohol use (30.56%), or opioid use disorders (27.78%). Despite the inclusion of treatment studies, this review showed that QoL measures were not used as predictors of outcome in any of the included studies.

Of the 108 studies, 68 (60.19%) included group comparisons to assess differences in QoL. These comparisons fell into four categories: (1) treatment group comparisons (38 studies, 60.19% of 65, comparing QoL outcomes between different treatment approaches or interventions (2) substance use (10 studies, 15.38% of 65), examining QoL differences across different substances of use, (3) mental health conditions (7 studies, 10.77% of 65), analyzing QoL variations based on co-occurring psychiatric diagnoses, and (4) other comparisons (10 studies, 15.38% of 65) (e.g., medical conditions, military status, stigma, and study completers vs. non-completers). According to the NIH quality assessment tool, of the articles included in the scoping review, 50.93% were rated as “good,” 48.22% were rated as fair, and 1.85% were rated as “poor.”

#### Application of QoL measures

Many studies using a G-QoL (*n* = 73) measure included participants using multiple substances (n = 29, 39.73%). Of the G-QoL domains that could be assessed, most of the studies only measured psychological (*n* = 55, 75.34%), physical (*n* = 55, 75.34%), and social (*n* = 47, 64.38%). Over half of the studies were conducted in an outpatient setting (*n* = 41, 56.16%), where participants received medical detox/medication management (*n* = 39, 53.42%). Among the studies examining differences in G-QoL between groups, most showed significant differences (60 of 73, 82.19%), with 26 studies showing differences between time points (43.33%) and 22 showing differences between treatment groups (36.67%).

Most of the studies using an HRQoL measure included participants reporting a primary substance: alcohol (44.12%) and opioids (35.29%). Of the HRQoL domains that could be assessed, most of the studies measured psychological (91.17%), physical (91.97%), and independence (26.47%) domains. Over half of the studies were conducted in an outpatient setting (21 of 34, 61.76%), where participants received medically assisted treatment (23 of 34, 67.65%). Among the studies examining differences in G-QoL between groups, most showed significant differences between treatment groups (7 of 34, 20.59%). Approximately 17.65% (6 of 34) of studies examining pre- and post-treatment differences in G-QoL showed a significant improvement in QoL. These results display similar effectiveness in detecting group differences but are less sensitive to changes than G-QoL, possibly due to the length of time and treatment condition.

#### Treatment setting

In an inpatient treatment setting***,*** the most used QoL measures were the WHOQOL-BREF (50.00%) and SF-36 (17.86%). For inpatient treatment, approximately 75.00% of the articles included medical assistance alone as the treatment for SUD. Over half of the studies included information on psychological (85.71%), physical (85.71%), and social (64.42%) domains related to QoL. Residential treatment centers mainly assessed for psychological (60%), physical (60%), and social and environmental (40%) domains of QoL.

Outpatient treatment settings utilized the WHOQOL-BREF (30.16%), SF-36 (17.46%), and EQ-5D (17.46%) questionnaires. Nearly half of the articles were at an outpatient center that utilized medical treatment (52.38%), and almost 26.98% included a psychotherapy intervention, suggesting that medically assisted treatment programs preferred HRQoL measures. Many of the studies included information on psychological (79.36%) and physical (77.77%) domains, and over one-third included information on the social domain (38.09%). For studies that included multiple treatment settings (e.g., inpatient, outpatient, residential, aftercare), all assessed psychological and physical domains, and two-thirds assessed social and environmental domains.

### Aim 2: QoL domains

Seven QoL domains were identified (e.g., psychological, physical, social, environmental, independence, security, and spiritual). Table 3 provides percentages for each domain and subdomain for aggregated QoL measures.

**Table 3:** Features of quality-of-life domains

Features of quality-of-life domains	Full sample	
	*n*	%
Psychological Domain (N = 86)		
Mental Health Symptoms	47	54.65
Emotions (positive & negative)	21	24.42
Emotional Impairment	10	11.63
Self-Esteem/Acceptance	10	11.63
Emotional Well-Being	8	9.30
Body Image	7	8.14
Cognition/Memory/Learning	7	8.14
Other: Creativity, Fulfillment, Impulsivity, Leisure	4	4.65
Physical Domain (N = 84)		
Physical Functioning	47	55.95
Pain	34	40.48
General Health	19	22.62
Energy/vitality	24	28.57
Activities of Daily Living	14	16.67
Mobility	11	13.10
Medication	5	5.95
Sleep	3	3.57
Appetite	1	1.19
Social Domain (N = 50)		
Personal Relationships	23	46.00
Social Functioning	23	46.00
Sexual Activity	7	14.00
Social Support	7	14.00
Relationship with Self	3	6.00
Environmental Domain (N = 36)		
Home Environment	11	30.56
Access to Services/ Health Care	4	11.11
Transportation	3	8.33
Access to Food	2	5.56
Community Environment	2	5.56
Independence Domain (N = 20)		
Ability to Engage in Usual Activities	19	95.00
Work	13	65.00
Self-Care	8	40.00
Freedom	4	20.00
Security Domain (N = 5)		
Physical Safety	5	100.00
Financial Security	4	80.00
Spiritual Domain (N = 4)		
Spirituality	4	100.00
Personal Beliefs	4	100.00
Philosophy on life	1	25.00

Percentages are calculated from the total number of times the domain was included, not out of the total number of articles

#### Primary domains (most assessed)

The most common domains assessed were psychological (80.56%) and physical (79.63%) domains, as measured by the WHOQOL-BREF, SF-36, and EQ-5D. The psychological domain evaluates mental health symptoms (i.e., “How much of a problem do you have with anxiety or depression today”) (EQ-5D), subjective ratings of positive (i.e., satisfaction) (WHOQOL-BREF), and self-report of impairment caused by emotions (SF-36). Additionally, subdomains included emotional well-being, body image, and cognition/memory/learning [29, 32, 37, 41, 42, 44, 45, 91].

QoL measures, such as the WHOQOL-BREF, SF-36, and EQ-5D, assessed the physical domain. The physical domain assessed symptoms such as general health, pain, and energy levels. Over half of the studies evaluated physical functioning (i.e., “Does your health limit you in moderate activities?”) (SF-36), individual pain ratings (i.e., “How often does pain impact your ability to enjoy daily activities?”) (Q-LES-Q) [134], and energy and vitality (i.e., “Do you have enough energy for everyday life?”) (WHOQOL-BREF). Additional subdomains included activities of daily living, mobility, medication, sleep, and appetite [22, 29, 37, 41, 42, 45, 51, 104, 118, 123].

#### Secondary domains

The next most common were social and environmental domains. The social domain was assessed in 46.30% of the studies, personal relationships (i.e., “How satisfied are you with your relationships?”) (WHOQOL-BREF), and social functioning (i.e., “How often do you feel isolated from others because of your health?”) (SF-36). Other subdomains included sexual activity, social support, and relationship with self. The environmental domain, assessed in 33.33% of the studies, references an individual’s work environment, living conditions, and access to education and healthcare (i.e., “How satisfied are you with the accessibility of health services?”) (WHOQOL-BREF). The remaining subdomains included transportation, access to food, and community environment [29, 41, 45, 118].

### Less frequently assessed domains

The independence, security, and spirituality domains of QoL were less frequently assessed. The independence domain was evaluated in 18.52% of the studies, including information on the individual’s ability to complete independent activities (i.e., “Do you feel capable of handling life’s responsibilities on your own?) (Q-LES-Q). The security domain was assessed in 4.63% of the studies, all assessing physical safety (i.e., “Do you feel protected from harm or danger in your living environment?”) and approximately half evaluating financial security (i.e., “How satisfied are you with your financial stability?”) (Q-LES-Q). The spiritual domain was assessed in 3.70% of the studies. All studies included information on satisfaction with their beliefs (i.e., “How satisfied are you with your spiritual life or sense of purpose?”) (QOLI) [135].

### Aim 3: patterns in demographic and cultural influences on QoL

Examining geographic distribution and publication timeframes revealed distinct patterns in QoL measure selection and application across region and over time. Geographic variations showed a strong preference (12 of 16, 75.00%) for the WHOQOL-BREF measure among studies conducted in Asian countries. Other countries demonstrated more variability in measure selection across general and HRQOL: North America: WHOQOL-BREF (31.43%), SF-36 (20.00%), EQ-5D (14.29%), SF-12 (13.29%), and the Q-LES-Q (14.29%); Europe: EQ-5D (35.53%), the SF-36 (23.40%), and the WHOQOL-BREF (17.02%).

### Aim 4: temporal trends in QoL measurement

Early studies published before 2013 displayed a variety in the usage of QoL measures, as the most used QoL measures were the SF-36 (23.81%), WHOQOL-BREF (14.29%), and the EQ-5D (19.05%), but included a range of 2.38–4.76% of other QoL measures (e.g., Multiple Sclerosis Quality of Life [136], Lancashire Quality of Life Profile [137], and the Quality-of-Life Scale [138]. More recent studies (2014-present) show a temporal trend of increased standardization over time, with a higher percentage of using the WHOQOL-BREF (42.47%), SF-36 (16.44%), and the EQ-5D (12.33%).

## Discussion

This study aimed to (1) characterize the types of QoL measures used in SUD treatment research, (2) examine how QoL domains are conceptualized and measured across different treatment settings and populations, (3) identify how patterns in how demographic factors, comorbidities, and cultural context influence QoL measurement and outcomes in SUD treatment, and (4) describe trends in QoL measurement approaches from 2000–2025 to understand how the field has evolved. Analysis of 108 studies published between 2000 and 2025 showed several key patterns: G-QoL was assessed more frequently than HR-QoL, psychological and physical domains were most evaluated out of the QoL domains, uneven geographical distributions of research and application of QoL measures, substantial gaps in demographic reporting, and concerning methodological trends. The following sections discuss these findings in more detail, considering their implications for improving QoL assessment in SUD treatment and identifying critical areas for future research.

### Aim 1: characterize the types of QoL measures used in SUD treatment research, including how these measures are defined and used

Results revealed patterns in measure selection and implementation across SUD treatment research. G-QoL was favored over HRQoL, and frequent measures included the WHOQOL-BREF (34.26%), SF-36 (20.37%), and EQ-5D (15.74%), suggesting a preference for broader QoL assessment in SUD treatment research. It is hypothesized that the G-QoL measures are preferred in SUD treatment due to the broad domains (e.g., social, psychological, environmental) that are associated with SUD and that drive recovery. However, the overlap between domains presents a significant challenge for measurement. For example, in the psychological domain on the WHOQOL-BREF, one category assessed was ‘self-esteem and acceptance’, which overlaps with the category ‘relationship with self’ under the social domain on the EQ-5D. This lack of clear domain differentiation complicates cross-study comparisons. Comparisons could lead researchers to draw conflicting conclusions about which treatment approaches most effectively improve specific QoL domains, which has the potential to misdirect clinical resources and treatment planning. Future research should focus on developing clear domain boundaries and examining whether the current domain structure adequately reflects the QoL experiences of individuals in SUD treatment.

### Aim 2: Examine how QoL domains are conceptualized and measured across different treatment settings (inpatient, outpatient, during treatment, after treatment) and populations

Outpatient studies demonstrated more robust methodological approaches, implementing more randomized controlled trials and longitudinal assessments, with approximately half including baseline-to-follow-up comparisons. However, only roughly one-third were assessed for differences between treatment groups, and very few were evaluated for other differences. Inpatient treatment centers relied more heavily on cross-sectional designs and did not use between-group comparisons. These difference are hypothesized to exist due to larger populations seen in outpatient settings that may lead to more rigorous longitudinal designs, longer follow-up periods, and reduced contextual bases compared to inpatient or residential treatment settings creating more robust research opportunities. Studies that employed longitudinal assessments did not capture differences between treatment time points (e.g., baseline, follow-up) and did not include comparisons between groups. These studies focused primarily on psychological, physical, and social domains, with medical assistance being the predominant treatment approach. Interestingly, inpatient settings demonstrated a greater assessment of social domains than outpatient settings (64.28% vs. 38.09%), despite outpatient treatment typically offering more opportunities for social interaction and community integration. This finding was unexpected and may reflect limitations in using currently available QoL measures, highlighting how future research is needed to examine whether the current domain structure adequately reflects the QoL experiences of individuals in SUD treatment. This difference in assessment of social domains is hypothesized to reflect the availability of QoL measure as well as diffuse social relationships seen in outpatient settings.

Importantly, across all treatment settings, the review found that QoL was exclusively used as an outcome measure, with no studies using QoL as a predictor of treatment outcomes. While it is hypothesized that using QoL domains to tailor treatment outcomes, QoL measures are designed as a responsive patient-report outcome measure to chart change over the course of treatment [139]. Significant gaps limit our understanding of how QoL influences treatment engagement, retention, and outcomes. Future research should investigate whether different treatment settings require different domain emphases, how the domains might impact treatment outcomes in various settings, how to implement more rigorous longitudinal assessment approaches in inpatient settings while accounting for their unique challenges, and whether initial QoL scores could serve as predictors of treatment success and how various treatment approaches impact QoL scores, potentially informing treatment planning an individualization of care.

### Aim 3: identify how patterns in how demographic factors and cultural context influence QoL measurement in SUD treatment

This review showed significant gaps in cultural representation and demographic reporting. Studies were heavily concentrated in Europe and North America (Europe: 43.51%; North America: 32.41%), with minimal representation from Asia, South America, Africa, and Oceania. This geographical imbalance is concerning given that problematic substance use is a global public health concern and the potential cultural variations impacting how QoL is conceptualized and experienced [15]. Beyond geographical representation, our review also revealed significant limitations in demographics characterization across studies. The lack of demographic reporting is equally problematic, with 72.22% of studies failing to report racial/ethnic demographics. This widespread omission of participant identities (e.g., race, ethnicity, socioeconomic status, immigration status) creates a significant blind spot in understanding how these factors influence QoL in SUD treatment. Moreover, additional key factors such as co-occurring physical and mental health conditions and housing status, all of which can significantly impact both SUD treatment and QoL were inconsistently reported and rarely examined concerning QoL outcomes. This gap is particularly concerning given the known disparities in SUD treatment access and outcomes across different demographic groups. Notably, the role of stigma was explored in one study [45], representing a critical gap in the literature. This oversight is particularly significant given that stigma can profoundly affect treatment-seeking, engagement, and outcomes [140]. It is hypothesized that this under-representation in the literature may reflect broader inequities in research funding and data collection, as well as omission of race and ethnicity in study design due to limited sample diversity or fear of marginalization of groups.

Given these findings, patterns in the association between QoL and demographic characteristics were not possible in this review. Future research should prioritize several areas: development and validation of culturally adapted QoL measures that account for diverse conceptualizations of well-being; systematic examination of how intersecting identities influence QoL experiences and treatment outcomes, investigation of how stigma impacts QoL across different treatment settings and populations, exploration of QoL assessment in correctional settings and other underrepresented treatment contexts, and analysis of how socioeconomic factors and co-occurring conditions moderate the association between treatment and QoL outcomes. Additionally, standardized reporting of demographic characteristics should be emphasized to facilitate a more comprehensive review of how cultural and demographic factors influence QoL in SUD treatment.

### Aim 4: Describe trends in QoL measurement approaches from 2000 to 2023 to understand how the field has evolved

When looking at trends between studies published between 2000–2013 and those after 2014, both periods predominantly include a G-QoL measure, such as the WHOQOL-BREF and EQ-5D, that looks at psychological, physical, and social domains. Studies published after 2014 had a higher rating of “good” quality assessments than articles published between 2000 and 2013. Notably, there was a decline in the majority of randomized control trials from 2000 to 2013 to 2014 -present and an increase in the majority of cross-sectional studies. In earlier research, there was a higher percentage of studies (69.44%) including QoL assessments between baseline and follow-up, compared to more recent studies where this number decreased to approximately 38.89%. These results highlight the inconsistent inclusion of diverse demographic data and the lack of in-depth longitudinal QoL assessments, which continue to limit understanding of how SUD treatment impacts QoL outcomes. Additionally, result trends revealed that studies published after 2014 had a higher probability of non-significant results than articles published before 2013, and studies published between 2000 and 2013 found a higher percentage of significance in QoL scores between-group comparisons. These trends suggest a need for a renewed emphasis on rigorous longitudinal assessment approaches while maintaining improved quality standards. However, any global standard for demographic reporting must retained be flexible to capture culture and context specific determinants of health disparities globally.

### Limitations

Several limitations of this scoping review should be considered. First, despite our comprehensive search strategy, we may have missed relevant studies published in languages other than English or indexed in databases we did not search. Second, our review was limited to peer-reviewed literature and may have missed relevant findings in gray literature, conference proceedings, or unpublished studies. Third, while we used a structured quality assessment tool, evaluating study quality involves subjective judgment, particularly when assessing methodological rigor across different treatment contexts and study designs. Fourth, our inclusion criteria focusing on clinical SUD diagnoses may have excluded studies examining QoL in individuals with substance use who did not meet full diagnostic criteria, potentially limiting our understanding of the full spectrum of substance use and QoL relationships. The inconsistencies in how QoL is measured and analyzed make comparing results across the different studies, settings, and cultures challenging. Regarding the article’s methodological quality, many articles lack information on co-occurring mental and physical health conditions and demographic information, which impedes the generalization of the results regarding QoL. The variability in SUD treatment availability, accessibility, and affordability across cultures may influence the feasibility of conducting treatment research, as well as the treatment outcomes for those who receive it, thereby limiting the generalizability of the findings. Lastly, research supports that established that SUD treatment should last a minimum of two years []141, thus this scoping review fails to look at individuals who are still receiving SUD treatment but have attained abstinence. This review fails to include individuals in early remission, and future research should consist of all phases of substance use treatment.

## Conclusions

This scoping review revealed considerable variability in QoL and SUD treatment research. The results revealed that many studies under-reported race, ethnicity, and cultural contexts, which is critical in adapting more culturally sensitive and patient-centered QoL measures. Additionally, the predominant focus on adult populations highlights the importance for clinicians to be proactive in addressing SUD in adolescent populations. Results also showed high variability in the definition of QoL domains and their standardization across treatment locations and geographic regions. This indicates the need for future research to explore the effect sizes and item responses of QoL measures in SUD treatment. Thus, this review highlights the need to operationalize QoL domains and assessment approaches in SUD treatment research to facilitate meaningful comparisons and enhance our understanding of treatment effectiveness, paving the way for improved patient care and treatment strategies and ultimately leading to better treatment outcomes.

## Data Availability

Data supporting the findings of this study are available from the corresponding author upon request.
